# Use of fly-ash slurry in backfill grouting in coal mines

**DOI:** 10.1016/j.heliyon.2017.e00470

**Published:** 2017-12-01

**Authors:** Ning Jiang, Jinhai Zhao, Xizhen Sun, Liyang Bai, Changxiang Wang

**Affiliations:** a State Key Laboratory of Mining Disaster Prevention and Control Co-founded by Shandong Province and the Ministry of Science and Technology, Shandong University of Science and Technology, Qingdao 266590, China; b College of Mining and Safety Engineering, Shandong University of Science and Technology, Qingdao 266590, China

**Keywords:** Environmental science, Safety engineering, Mechanical engineering

## Abstract

Cave backfill grouting implies grouting of the caving rock mass prior to it being compacted. The filling materials strengthen the caving rock and support the overlying strata to achieve the purpose of slowing down the surface subsidence. The broken roof will fail and collapse during mining operations performed without appropriate supporting measures being taken. It is difficult to perform continuous backfill mining on the working face of such roofs using the existing mining technology. In order to solve the above problems, fly ash and mine water are considered as filling materials, and flow characteristics of fly-ash slurry are investigated through laboratory experiments and theoretical analyses. Laws governing the diffusion of fly-ash slurry in the void of caving rock masses and in the void between a caving rock mass and a basic roof are obtained and verified. Based on the results obtained from the above analyses and actual conditions at the Zhaoguan coal mine, Shandong Province, China, a cave backfill grouting system of the hauling pipeline is developed and successfully tested at the 1703 working face in the Zhaoguan coal mine. The results demonstrate that a filling rate of 43.46% is achieved, and the surface subsidence coefficient of the grouting process is found to be 0.475. Compared to the total caving method, the proposed system is found to achieve a reduction rate of 40.63%. This effectively helps in lowering the value of the surface subsidence coefficient. Fly ash and mine water, considered as primary materials in this study, also play a significant role in improving the air quality and water environment.

## 1 Introduction

Coal accounts for 68.5% of the primary energy consumption and forms the main source of energy in China. It is highly unlikely that this situation would change in the near future. The total coal in the three state-owned coal mines in the Shandong province alone weighs approximately 13.8 billion tonnes, nearly 9 billion tonnes of which remain under residential buildings and account for nearly 60% of the recoverable energy reserves. In this scenario, backfill mining technology offers the best means to extract the coal that is buried under residential buildings. This technology has the advantages of high recovery rate, controlled movement of the overlying strata, efficient reduction of surface subsidence, and being potentially non-hazardous to the environment in terms of solid-waste treatment. The filling modes currently employed in this technique include gangue backfilling behind a mechanised supporter [1, 2, 3, 4, 5], paste backfilling [6, 7, 8, 9, 10], high water backfilling [11, 12, 13, 14], and separation layer grouting [15, 16, 17]. These modes a vital role in the reducing surface subsidence and controlling the movement of overlying strata. However, the existing filling and mining technology requires filling of the gob area as a prerequisite prior to the caving of the immediate roof. As such, the roof must possess above-average stability to ensure optimum filling. Broken joints in the roof possess poor integrity and lack structural stability. They may, therefore, fail and collapse during mining operations performed without appropriate supporting measures being taken. It is, therefore, difficult to perform continuous backfill mining on the working face of such roofs using the existing mining technology. Based on the above characteristics, Zhang put forward the method of wide-strip filling full pillar mining [18, 19], which proceeds in two stages—wide-strip mining and full pillar extraction. In this method, the wide strip is used to support the immediate roof to ensure adequate filling time. However, the mining process is complicated. Scientists from Germany and Poland proposed grouting in the caving areas of longwall mining [20, 21, 22]. This technology focuses on filling the void between the waste rocks before the void is compacted. The filling body is cemented with the gangue to support the roof. However, filling is carried out after working face mined completely, and broken rocks in gob has been compressed. As a result, the filling space ends up being smaller and the control effect of filling body is, therefore, limited. Based on the above characteristics, this study proposes a new surface subsidence technology—hauling pipeline backfilling for cave backfill grouting. Key features of the proposed technology include void grouting of the caving rock mass, filling of caving rock gaps with the hauling pipeline behind the hydraulic support, reduction in compression rate and strengthening of broken rocks, improvement in bearing performance thereby maintaining control over slowly moving overlying strata, and reduction in surface subsidence. The proposed technique is cost effective and requires neither transformation of the hydraulic support nor sealing of the mined-out area. Moreover, the proposed technique ensures simultaneous realisation of, both, filling and mining operations as well as safe disposal of solid waste such as fly ash which has great significance in reducing the occupation of cultivable land and environmental protection.

## 2 Geological setting

**Fig. 1:**
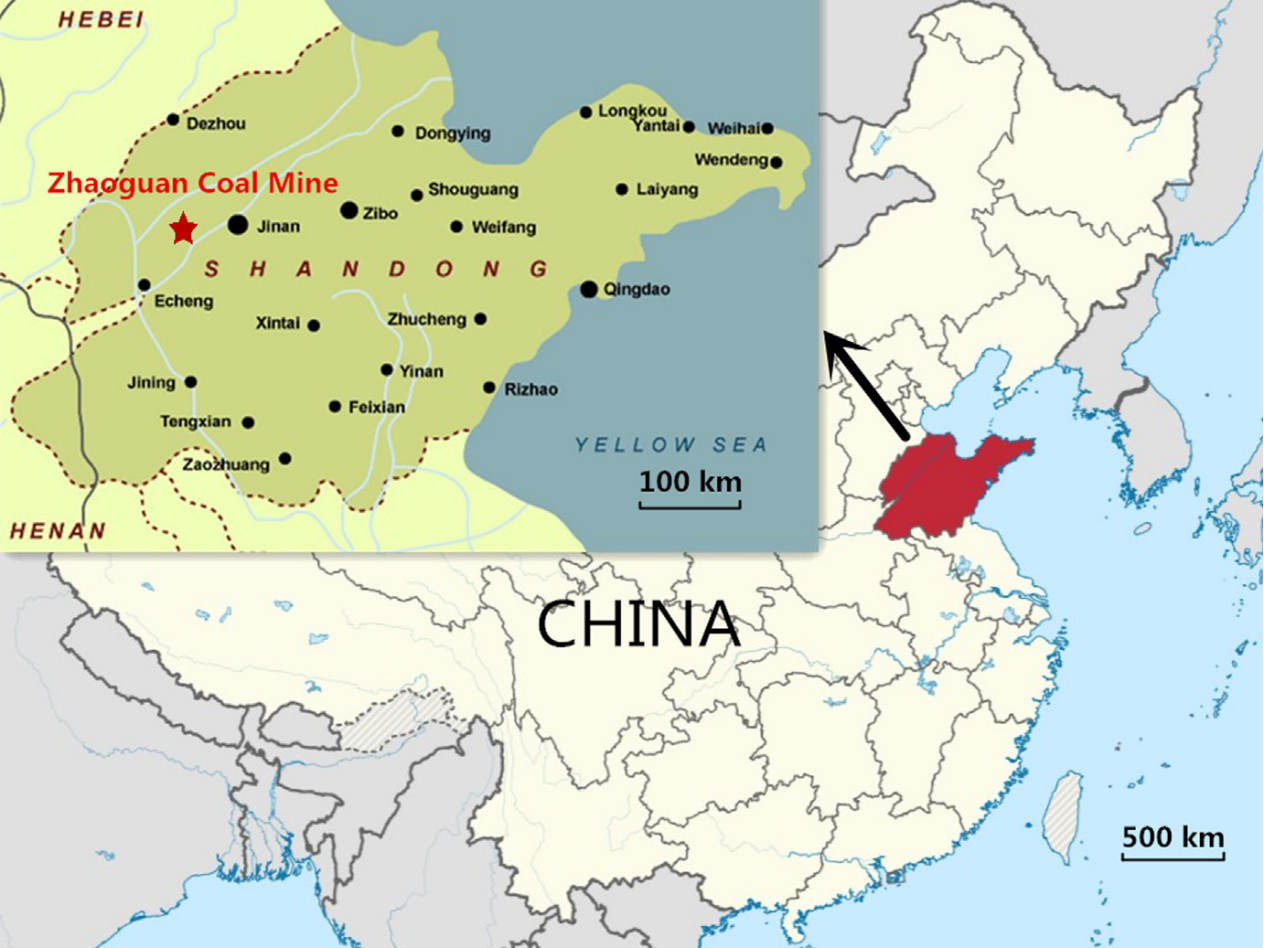
The location of Zhaoguan coal mine.

Zhaoguan coal mine lies in south − west Qihe, Shandong Province, China (as shown in Fig. 1). The 7# coal seam comprises 1703 working face, and is located in the upper part of the Taiyuan formation. NO. 1 limestone (the first layer limestone from top to bottom) is located above the 7# coal seam, and the distance between the two is 14.93–25.30 m (average 18.96 m). The distance between the 5# and 7# coal seams is 38.50–47.10 m (average 42.52 m). Similarly, the distance between the 7# and 8# coal seams is 9.79–16.57 m (average 13.33 m). The 1703 working face is located at a depth of 415 m below ground, and the coal seam has an 80−91° northward tilt and a relatively flat dip angle of 3−5°. Thickness of the coal seam is 0.80–1.20 m, while the average coal thickness is 0.95 m. The recoverable index of the coal seam is a parameter that represents the proportion of the recoverable coal seam in the evaluation area and has a value of 1 in the present case. Likewise, the variation coefficient of coal thickness is a parameter that measures coal thickness off the average thickness. It has a value of 20% for the present case. The coal seam macro component is of the semi-bright type, with a small amount of coal and charcoal silk belt.

**Fig. 2:**
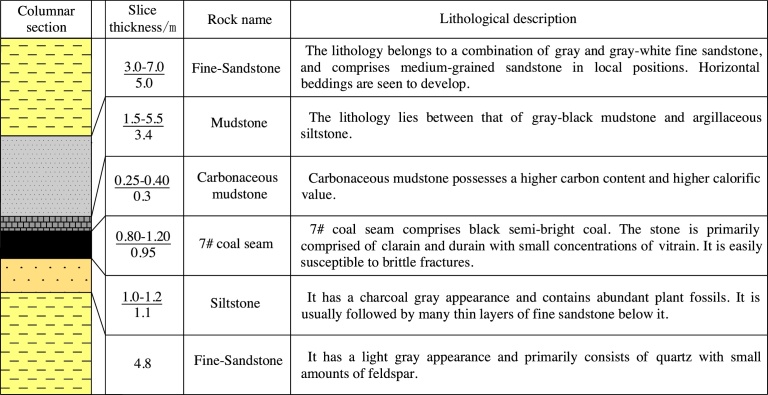
Column map of the 1703 working face at the Zhaoguan coal mine.

The immediate roof of the 1703 working face is comprised of mudstone (Fig. 2), its thickness varying between 1.5–5.5 m, with an average of 3.4 m. The basic roof of the 1703 working face is made of fine sandstone, the thickness of which varies from 3 to 7 m, i.e., an average thickness of 5 m. There exists a layer of carbonaceous mudstone at the top of the coal seam. The thickness of this layer varies from 0.25 to 0.4 m, with an average of 0.3 m. At the immediate bottom lies siltstone with a thickness varying from 1 to 1.2 m (an average of 1.1 m). The basic bottom is again comprised of fine sandstone with a thickness of 4.8 m.

The 1703 working face is a fully mechanised longwall working face. The total trend length of this working face is 960 m, and the tendency length is 215 m. The mining height of the working face is 1.2 m.

## 3 Materials & methods

### 3.1 Materials

**Fig. 3:**
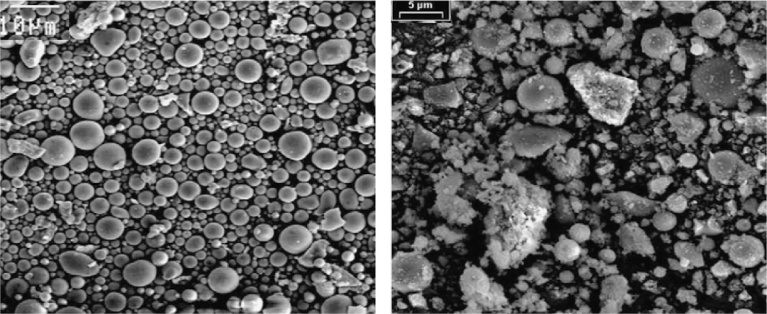
SEM image of fly ash with different magnifications (Left) 10-μm scale and (Right) 5-μm scale.

Fly-ash slurry was chosen as the filling material in this study. Fly-ash slurry is a mixture comprising mine water and fly ash mixed in an 8:10 proportion. Fly ash is obtained primarily from thermal power plants in the vicinity of the mining area. A scanning electron microscope (SEM) was used to study the particle-size distribution, and the results are plotted in Fig. 3. Fly ash consists of oxides such as SiO_2_, Al_2_O_3_, FeO, Fe_2_O_3_, CaO, and TiO_2_. Mine water is obtained from the pump room under the shaft of the Zhaoguan coal mine. There exist large amounts of suspended matter in mine water owing to its long-term contact with coal seam and rock and the influence of mining activities. It is found that there exist large amounts of sulphate and ferrous ions in mine water thereby contributing to its very low pH value.

### 3.2 Experimental study on flow and diffusion laws of fly-ash slurry

#### 3.2.1 Permeation grouting theory

**Fig. 4:**
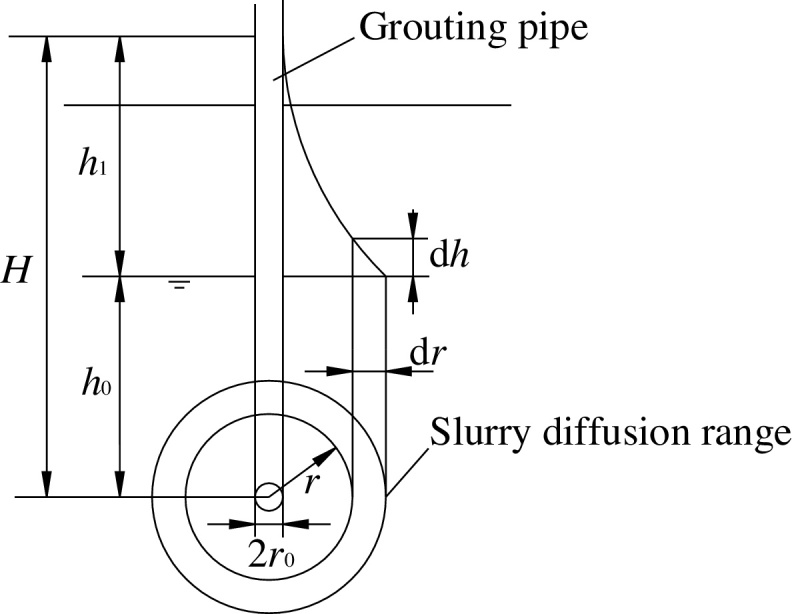
Schematic of the spherical diffusion theory.

An efficient method of diffusing fly-ash slurry in cave zones is permeation grouting. The penetration formula for slurry in sand layers was derived by Maag in 1938 [23]. Two assumptions are proposed in the context of fly-ash slurry—(1) The grouting source is a point source; (2) Fly-ash slurry is a Newtonian fluid. Once these assumptions are met, the slurry is found to diffuse in the ball (as shown in Fig. 4).

According to the Darcy's law, one may get

(1) $$Q=AK_{c}Jt$$

(2) $$J=\frac{\text{d}h}{\text{d}r}$$

(3) $$K_{c}=\frac{K}{\beta }$$

(4) $$A=4\pi r^{2}$$

Eqs. (2), (3) and (4) are introduced into Eq. (1), and according to the boundary conditions, we can deduce Eq. (5) and (6) from Eq. (1).

(5) $$H-h_{0}=\frac{Q\beta }{4\pi Kt}\left(\frac{1}{r_{0}}-\frac{1}{r_{1}}\right)$$

(6) $$Q=\frac{4\pi Kt(H-h_{0})}{\beta \left(\frac{1}{r_{0}}-\frac{1}{r_{1}}\right)}$$

It's known that $H-h_{0}=h_{1}$, $Q=\frac{3}{4}\pi r_{1}^{3}\cdot \phi$. In view of the fact that $r_{1}$ is much larger than $r_{0}$, namely $\frac{1}{r_{0}}-\frac{1}{r_{1}}\approx \frac{1}{r_{0}}$, so the above Eq. (5) and (6) can be simplified respectively as Eq. (7) and (8)

(7) $$h_{1}=\frac{r_{1}^{3}\beta \phi }{3Ktr_{0}}$$

(8) $$r_{1}=\sqrt[{3}]{\frac{3Kh_{1}tr_{0}}{\beta \phi }}$$

where Q is the grouting capacity per unit time (cm^3^/s); A is infiltrating area of slurry (cm^2^); J is hydraulic gradient; t is the grouting time (s); $K_{c}$ is permeability coefficient of the slurry in the soil layer (cm/s); K is the permeability coefficient of the soil layer (cm/s); β is the ratio of size viscosity and water viscosity, $\beta =\frac{\mu_{g}}{\mu_{w}}$; $r_{0}$ is radius of the filling pipe (cm); r, $r_{1}$ is diffusion radius of slurry (cm); ϕ is the porosity of the soil; $h_{0}$ is pressure head of underground water (cm); $h_{1}$ is pressure head of grouting (cm); H is sum of pressure head of underground water and grouting (cm); $\mu_{g}$ is coefficient of kinetic viscosity of slurry (mPa·s); $\mu_{w}$ is coefficient of kinetic viscosity of water (mPa·s); dh is the change of pressure head of grouting (cm); dr is the change of diffusion radius of slurry (cm).

#### 3.2.2 Test plan and equipment

In order to understand the laws governing the flow of fly-ash slurry in the caving zone, a test was conducted in the laboratory to investigate the influence of particle size and position of the grouting hole on the flow properties of fly-ash slurry. Two position of grouting hole (at the top and bottom of the caving zone) were considered along with three particle size grades— I (particle size of 9.5–16 mm), II (particle size of 16–20 mm), and ¢ó (particle size of 20–25 mm)—to account for the effect of particle size. A total of 6 group tests were conducted as summarised in Table 1. In consideration of the dispersion of the data, every group test was repeated three times, and average value of three times was taken as the test result.

**Table 1:** Test plan to investigate the laws governing the flow of fly-ash slurry in the caving zone.

Test number	Influencing factor		Test number	Influencing factor	
	Particle size grade	Position of grouting holes		Particle size grade	Position of grouting holes
P1	I	Top	P4	I	Bottom
P2	II	Bottom	P5	II	Top
P3	III	Top	P6	III	Bottom

Note: A. “Bottom” indicates position of the grouting pipe close to the floor; B. “Top” indicates its position from the top of the gangue collapse.

**Fig. 5:**
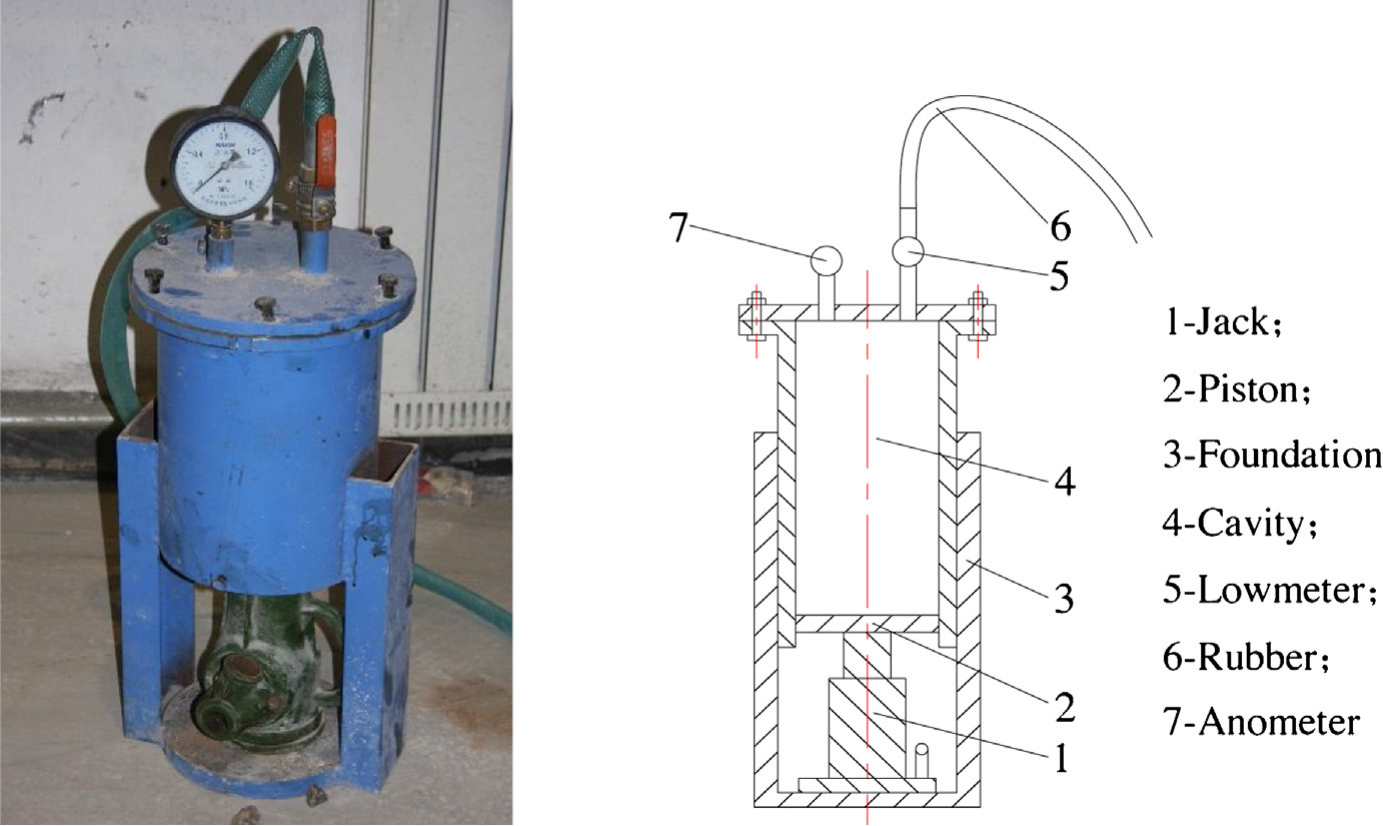
(Left) Outer appearance of grouting pump, (Right) Schematic of the grouting pump.

**Fig. 6:**
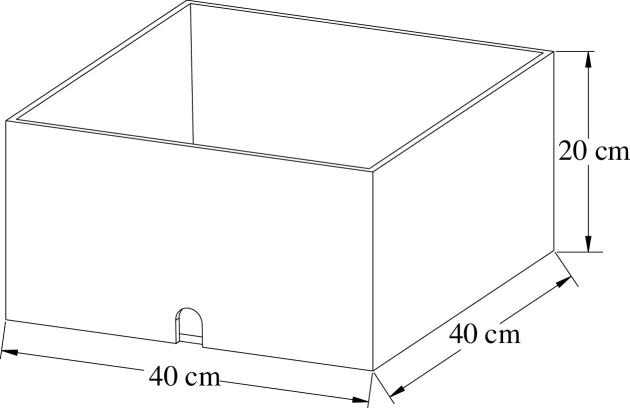
The test storehouse.

A manual filling grouting pump was considered as the experimental power source (Fig. 5). A test storehouse, comprising a cuboid with dimensions of 40 × 40 × 20 cm (Fig. 6), is used for holding the gravel. Grouting methods used are classified into two categories. In the first category, grouting is performed with the grouting pipe close to the floor; in the second category, the grouting operation is performed from the top of the gangue collapse. When a large amount of leakage was observed from the bottom of the test storehouse (grouting close to the floor) or the surface of the powdered coal slurry was found to spread to the edge of the test box (grouting in the upper part of the gangue), the grouting operation was said to have met the design requirements, and the test was terminated.

#### 3.2.3 Analysis of experimental results

Over the course of the above experiment, the accumulated flow quantity, filling ratio, grouting pressure, and depositing angle (i.e., the angle formed between the final deposit and the horizontal plane when the grouting hole is in the lower part) were regularly monitored. These monitored values are summarised in Table 2.

**Table 2:** Values of the monitored variables over the duration of the laboratory experiment.

Particle size grade	Monitored variable	Position of grouting holes							
		Top				Bottom			
		1	2	3	Average	1	2	3	Average
I	Filling capacity (L)	3.84	3.71	3.86	3.80	5.51	5.43	5.37	5.47
	Filling ratio (%)	62.54	60.42	62.87	61.89	89.74	88.44	87.46	89.09
	Grouting Pressure (MPa)	––	––	––	––	0.17	0.19	0.17	0.18
	Depositing angle (°)	––	––	––	––	53.75	54.12	55.07	54.31

II	Filling capacity (L)	4.32	4.47	4.39	4.39	5.79	5.68	5.84	5.77
	Filling ratio (%)	61.98	64.13	62.98	62.98	83.07	81.49	83.79	82.78
	Grouting Pressure (MPa)	––	––	––	––	0.14	0.17	0.16	0.16
	Depositing angle (°)	––	––	––	––	49.31	51.04	50.48	50.28

III	Filling capacity (L)	4.93	5.11	5.02	5.02	5.87	5.93	5.76	5.85
	Filling ratio (%)	63.04	65.35	64.19	64.19	75.06	75.83	73.66	74.81
	Grouting Pressure (MPa)	––	––	––	––	0.13	0.15	0.12	0.13
	Depositing angle (°)	––	––	––	––	45.13	46.32	44.74	45.40

Note: A. Pressure values quoted in Table represent the maximum filling pressure; B. When the grouting hole is at top, fly-ash slurry is not found at the bottom of the test box after the grouting is finished, and the final form of the silt-coal slurry is an inverted cone under the condition of. At this instant, its depositing angle is not measured. C. “1”, “2” and “3” represent the first, the second and the third experiment of each group test respectively.

**Fig. 7:**
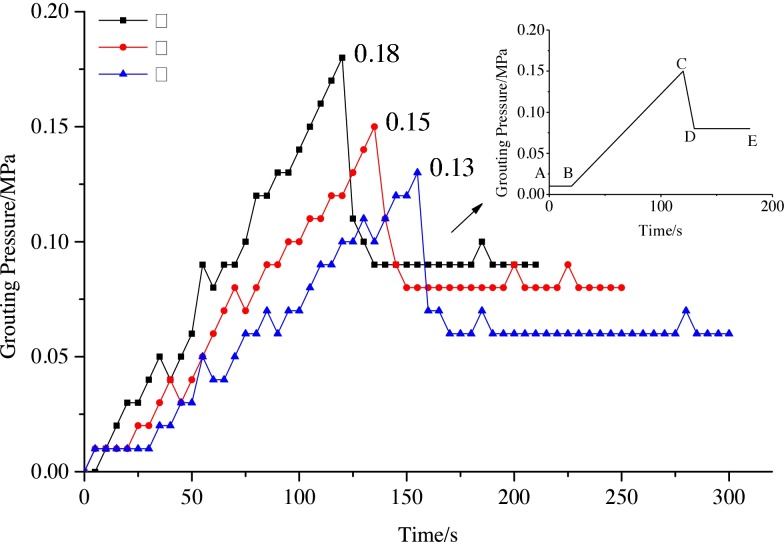
Grouting pressure-time relationship for three particle size grades.

The variation in grouting pressure with time for three particle size grades is depicted in Fig. 7. As can be realised from the figure, the variation in grouting pressure can be divided into four phases. During low operation phase (Segment AB), the grouting pressure-time curve is essentially a horizontal line representing the filling operation using fly-ash slurry through the grouting pipeline. In this phase, the operation resistance is constant and relatively small. Next, during the linear growth phase (Segment BC), the grouting pressure rises as a linear function of time. This stage represents the flow of fly-ash slurry in the waste rock. In this phase, an increase in powder coal slurry leads to an increase in grouting resistance. The third phase (Segment CD) represents a sharp decrease wherein the grouting pressure is restored to a smaller value in a short period of time. Gangue move under the grouting pressure, and form a grouting channel. During the final stable running phase (Segment DE), the grouting pressure–time curve once again takes the form of a horizontal line. Form a grouting channel between the grouting pipe and the grouting resistance remains essentially constant. The void volumes corresponding to the three particle sizes in the simulation box are *V_Q_*_I_ = 6.14 L, *V_Q_*_II_ = 6.97 L, and *V_Q_*_III_ = 7.82 L. *V_Q_*_I_, *V_Q_*_II_, *V_Q_*_III_ are the void volumes between the gravel of particle size grade I, II and III respectively. The larger the particle size, the larger is the equivalent diameter of the void formed. Large particle size also leads to a reduction in the maximum and stable values of the grouting pressure, its changed trend is Consistent with the law of equation (7). The grouting pressure curve demonstrates small fluctuations and a large particle material flow in grouting pipe during the test, and the near side of the pump pressure is far greater than the far pump for measuring pressure, what can be determined is caused by the uneven fly-ash slurry, the pressure fluctuation amplitude curve can be used as a fly ash monitoring uniformity index.

**Fig. 8:**
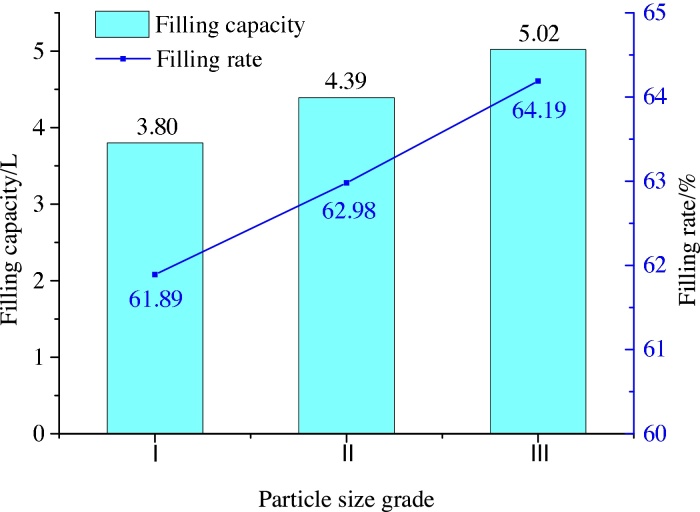
Variation of filling capacity and filling rate with particle size with the grouting pipe located at the top.

**Fig. 9:**
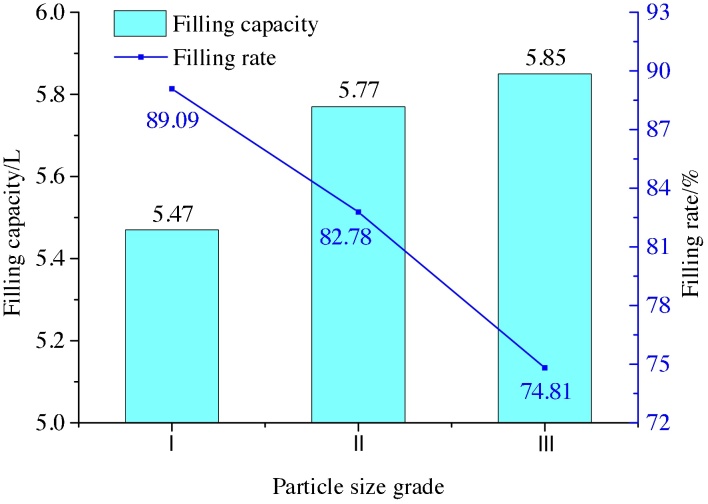
Variation of filling capacity and filling rate with particle size with the grouting pipe located at the bottom.

Figs. 8 and 9 below depict the variation in filling capacity and filling ratio with particle size for the two positions of the grouting pipe.

As seen in Figs. 8 and 9, the filling amount increases with increase in particle size, its changed trend is consistent with the law of equation (6). When the grouting hole is located at the top, the fly-ash slurry diffuses completely under the action of gravity, and the filling ratio increases with particle size. When the grouting hole is located at the bottom, the diffusion of fly-ash slurry occurs under the action of pump pressure. In this case, with an increase in particle size, the fly-ash slurry stacking angle and filling rate are found to decrease. For all particle sizes, the filling amount and filling rate are found to be greater when the grouting hole is near the bottom of the storehouse than when it is at the top. Therefore, this lower location of the grouting hole was preferred for real-world applications of the proposed technique.

## 4 Design

### 4.1 Slurry hauling pipeline backfill grouting process

**Fig. 10:**
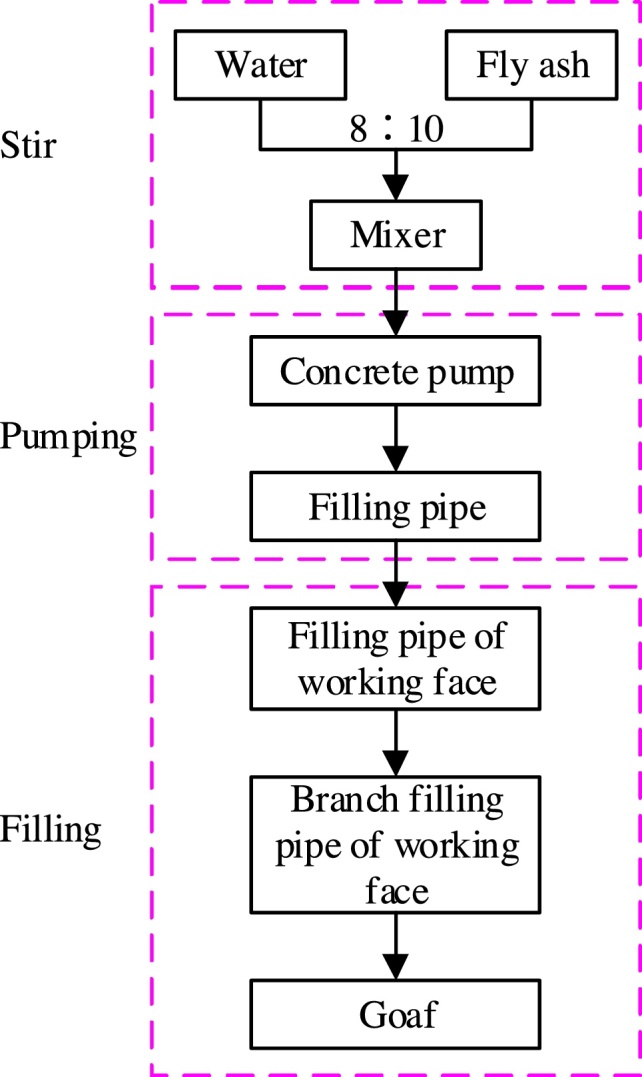
Flow diagram of fly-ash slurry hauling pipeline backfill grouting process.

The slurry hauling pipeline backfill grouting process can divided into three parts—stirring, pumping, and filling (as shown in Fig. 10).

Stirring: Mixing water and fly-ash thoroughly in a mixer in a certain proportion (8:10 by mass).

Pumping: Driving fly-ash slurry to the hopper of the concrete pump. The slurry is then transported to the working face through the concrete pump and filling pipe.

Filling: The slurry is finally carried to the goaf by the filling and branch filling pipes of the working face.

### 4.2 Slurry hauling pipeline backfill grouting system

The proposed slurry hauling pipeline backfill grouting system consists of three subsystems—stirring, pumping, and filling.

**Fig. 11:**
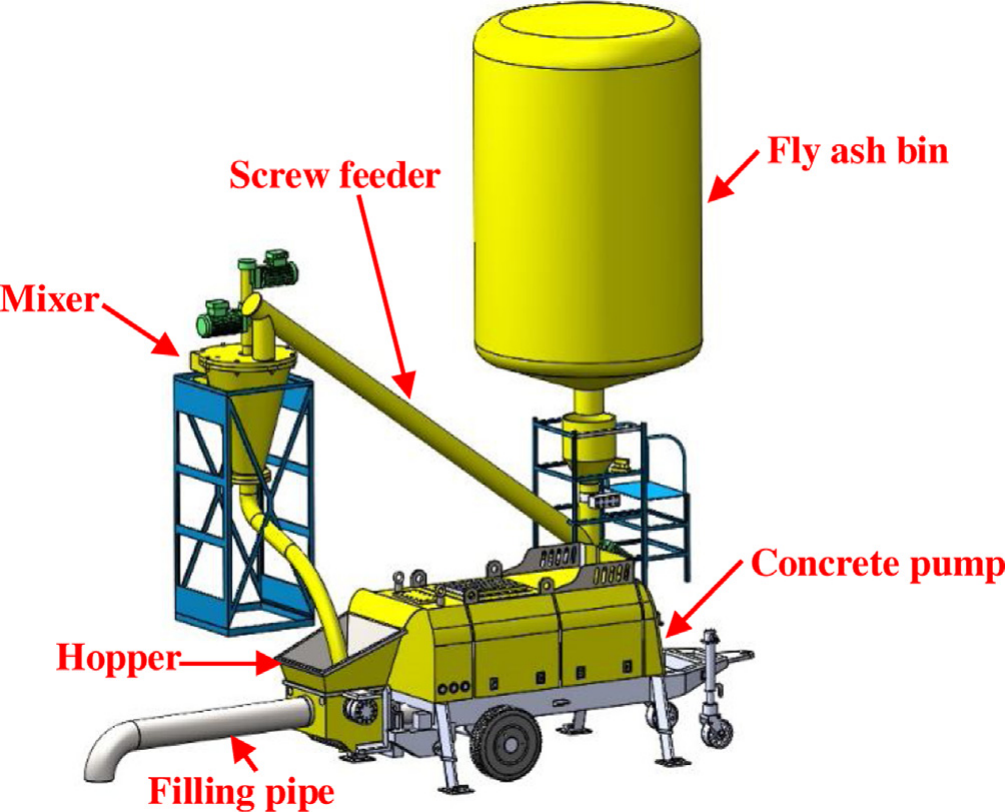
Stirring and pumping subsystems of the slurry hauling pipeline backfill grouting system.

The stirring subsystem mainly includes fly-ash bin, screw feeder, and mixer (Fig. 11). The fly-ash bin is used to stockpile the fly ash. Fly ash stockpiled in fly-ash bin is then brought into the mixer through the screw feeder. Fly ash and mine water are then fully mixed inside mixer in order to form fly-ash slurry.

**Fig. 12:**
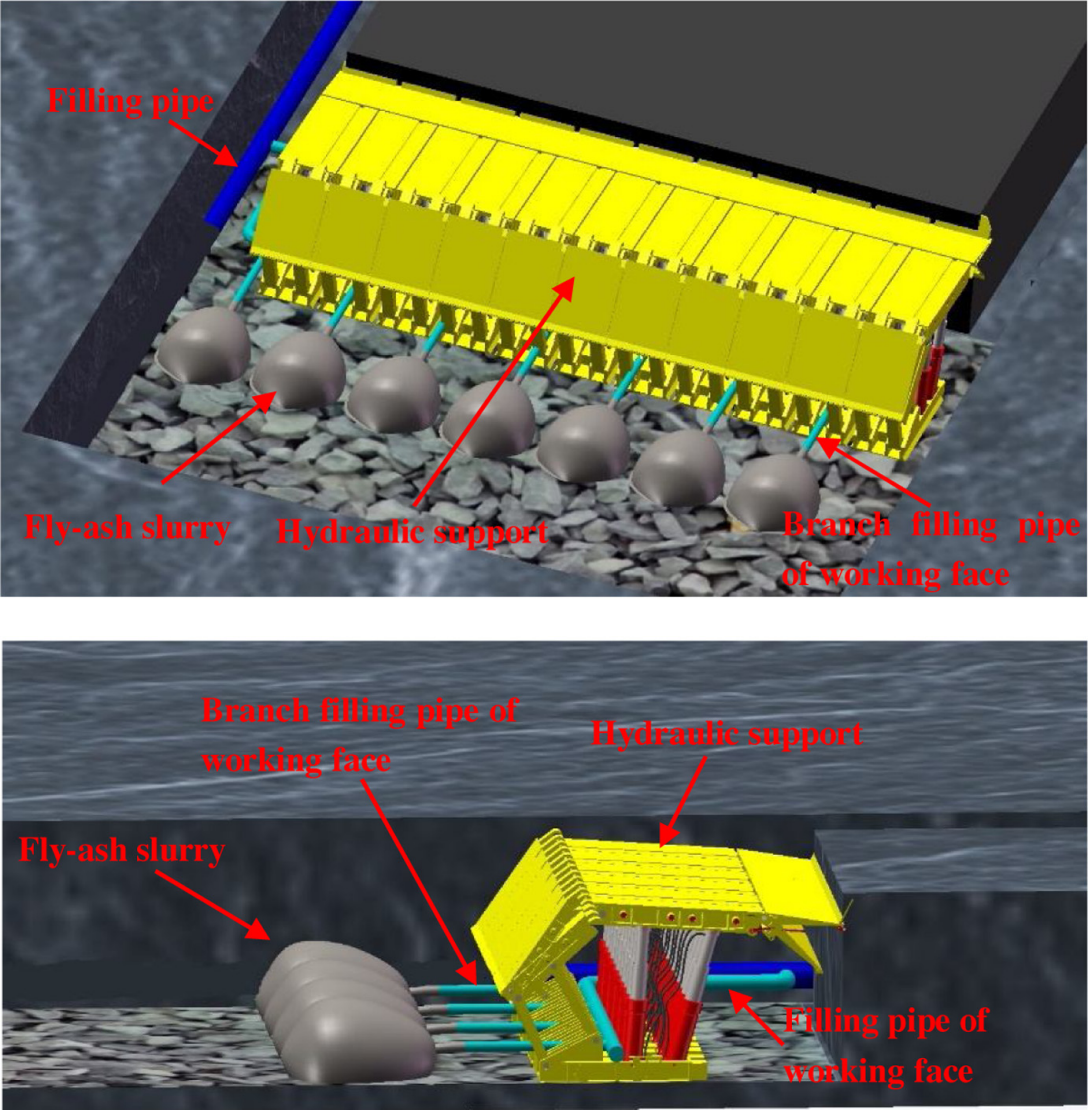
(Up) Back view of filling subsystem, (Down) Side view of filling subsystem.

The pumping subsystem comprises a concrete pump and filling pipe (Fig. 12). Fly-ash slurry prepared in the stirring system is driven to the hopper of the concrete pump and transported to the filling working face far away from stirring system.

The filling subsystem includes the filling and branch filling pipes of the working face (Fig. 12). The filling pipe is laid along tendency of the working face, and its length is approximately equal to tendency length of the working face. The branch filling pipe of working face is laid along trend of the working face, and its length varies between 10 to 20 m. One end of the branch filling pipe is connected to the filling pipe of working face.

## 5 Example

### 5.1 Filling system layout

**Fig. 13:**
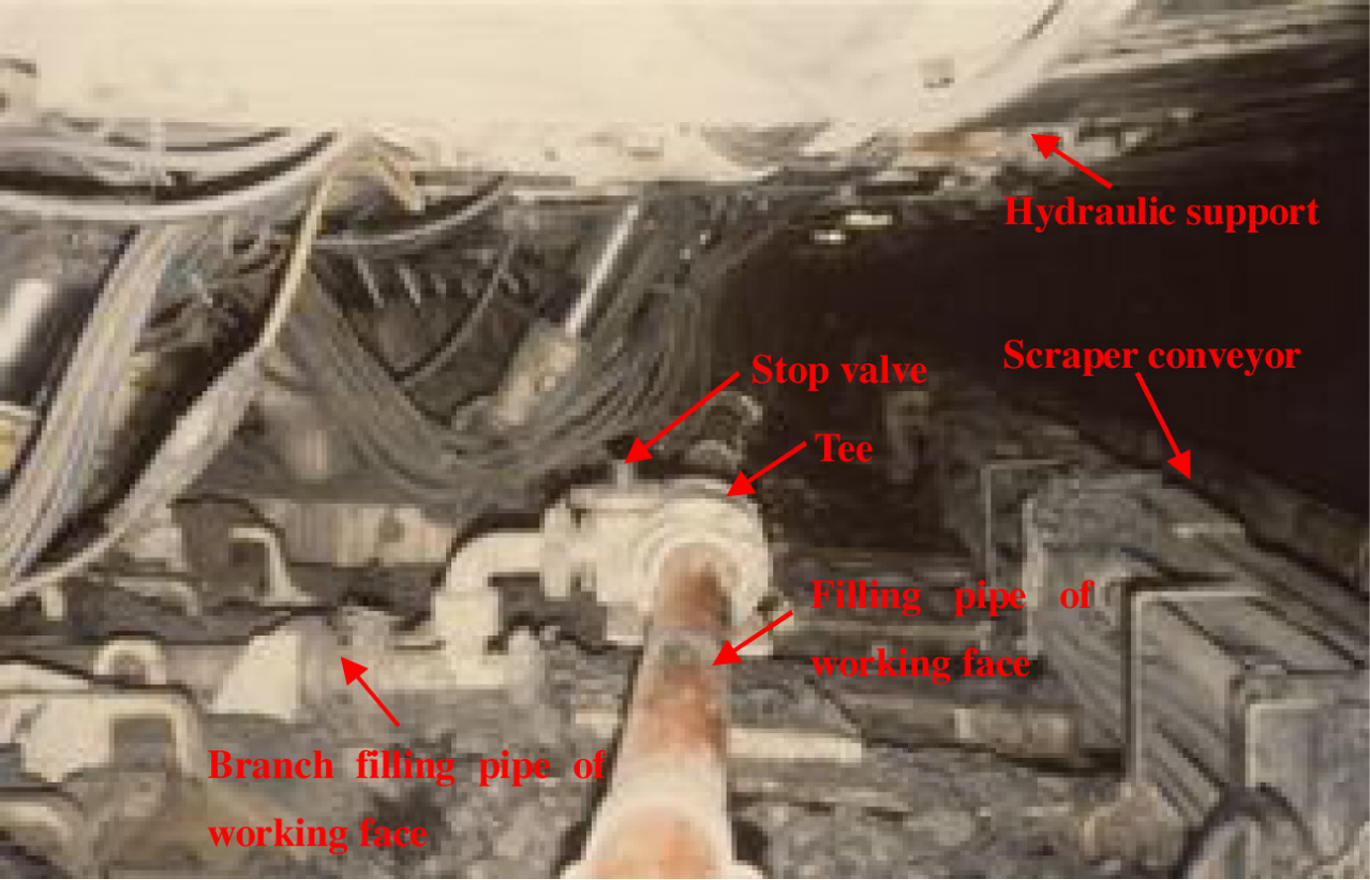
Filling subsystem of slurry hauling pipeline backfill grouting system.

An industrial test of the proposed fly-ash slurry hauling pipeline backfill grouting was carried out at the 1703 working face in Zhaoguan coal mine (as shown in Fig. 13). The whole filling system was located underground. The stirring and pumping subsystems were located at the working upper district station. The filling pipe (The pipe model is DN150) was laid up to the 1703 working face along the district raise and rail entry. The filling pipe of the working face was laid along the tendency of working face, and its length was measured as 215 m. A total of 15 tees were fitted on the filling pipe at intervals of 15 m. The branch filling pipe of the working face was connected to filling pipe through the 15 tees, and its length was 10 m. A stop valve and a flowmeter are installed on the branch filling pipe of the working face.

### 5.2 Filling process

The stepwise procedure to be followed during the fly-ash slurry hauling pipeline backfill grouting process are as follows.

(1) Open the stop value of the branch filling pipe of the working face on the lowest side.
(2) Close the stop value, and open another stop value according following the sequence from the low side to the high side.
(3) Repeat step two until the filling process of all 15 stop values are completed.
(4) When the working face mining proceeds forward by 10 m, repeat steps 1 through 3.
(5) Repeat step four until the working face has been mined completely.

A single filling cycle includes steps 1–3, as listed above. The filling volume of every branch filling pipe of the 1703 working face for the first six cycles is listed in Table 3. The filling rate is the ratio of filling volume to mining volume. Owing to the fact that the overlying strata around the open-off cut cave was not sufficient, the filling rate corresponding to the first filling cycle is only 22.40%, which is much smaller compared to values corresponding to other cycles. The average filling rate of the 1703 working face was determined to be 43.46% (the first filling cycle not taken into consideration).

**Table 3:** Filling volumes of the branch filling pipes of the working face and filling rates for first six filling cycles.

Filling cycle	Filling volume of each branch filling pipe of working face (m^3^)															Total filling volume (m^3^)	Filling rate (%)
	1#	2#	3#	4#	5#	6#	7#	8#	9#	10#	11#	12#	13#	14#	15#		
1	52	36	37	36	33	35	37	40	39	34	39	38	39	41	40	576	22.40
2	110	83	86	90	83	95	92	83	77	87	88	91	80	75	80	1300	50.44
3	91	79	75	81	79	85	90	78	74	74	81	75	69	75	79	1185	46.03
4	84	74	77	79	82	78	75	71	66	64	65	69	74	73	69	1100	42.78
5	81	70	67	68	68	72	68	67	64	60	63	68	60	73	78	1027	39.76
6	82	71	70	68	66	63	57	60	62	68	66	69	65	58	63	988	38.28

Note: A. The mining volume of every filling cycle is 2580 m^3^ (1.2 m × 10 m × 215 m); B. “1#”, “2#”, …, “15#” are the number of each branch filling pipeline of working face.

### 5.3 Effectiveness of reducing subsidence

**Fig. 14:**
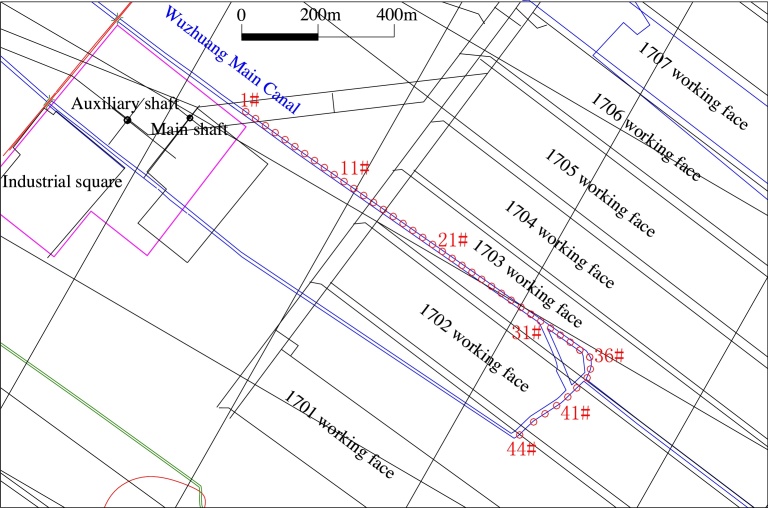
Monitoring scheme of surface rock movement.

The purpose of fill mining is to exercise control over surface subsidence. In order to evaluate the effectiveness of reducing subsidence during the fly-ash slurry hauling pipeline backfill grouting process, ground deformation observations were carried out on the surface of the 1703 working face. Two measuring lines (trend and tendency measuring lines) were laid along the Wuzhuang main canal (as shown in Fig. 14). The length of the trend measuring line was 1080 m, and the number of measuring points was chosen to be 36. The tendency measuring line was 240 m in length with 8 measuring points. The distance between the measuring points in both cases was 30 m.

Based on ground deformation results monitored over a long time, the maximum surface subsidence of the 1703 working face was found to be 0.57 m, and the subsidence coefficient was determined as 0.475. Subsidence coefficient is defined as the ratio between the maximum subsidence and mining thicknesses. According to the ground deformation monitoring results of the 1705 and 1706 working faces, the maximum subsidence is 0.96 m, and the subsidence coefficient is 0.80 under conditions of cave mining. Through comparison, it can be realised that the fly-ash slurry hauling pipeline backfill grouting process is well capable of controlling the movement of overlying strata thereby effectively reducing subsidence. The coefficient of subsidence reduction was found to be 40.63%, and represents ratio of the difference between the maximum subsidence under fill mining and cave mining to the maximum subsidence under cave mining.

## 6 Conclusions

On the basis of analyses and research concerning the advantages and disadvantages of existing filling technologies, a new filling technique called the fly-ash slurry hauling pipeline backfill grouting has been proposed in this paper. The process and system of the slurry hauling pipeline backfill grouting have been introduced and thoroughly explained in the foregoing sections. Results obtained from a real-world on-field application of the proposed technique demonstrate that the fly-ash slurry hauling pipeline backfill grouting process is capable controlling the movement of overlying strata thereby effectively reducing surface subsidence. The filling rate of the 1703 working face was found to be 43.46% with coefficient of subsidence reduction equal to 40.63%.

## Declarations

### Author contribution statement

Ning Jiang: Conceived and designed the experiments; Performed the experiments; Wrote the paper.

Jinhai Zhao: Performed the experiments; Analyzed and interpreted the data.

Xizhen Sun: Analyzed and interpreted the data.

Liyang Bai: Contributed reagents, materials, analysis tools or data.

Changxiang Wang: Performed the experiments.

### Funding statement

This work was supported by the National Natural Science Foundation of China (No. 51574159, 51704152), Shandong Province Natural Science Foundation (No. ZR2014EEM001, ZR2017BEE001), Shandong Natural Science Outstanding Youth Foundation (No. JQ201612).

### Competing interest statement

The authors declare no conflict of interest.

### Additional information

No additional information is available for this paper.
